# Qualitative insights into general practitioners views on polypharmacy

**DOI:** 10.1186/1471-2296-11-65

**Published:** 2010-09-15

**Authors:** Sibyl Anthierens, Anneleen Tansens, Mirko Petrovic, Thierry Christiaens

**Affiliations:** 1 Vaccine & Infectious Disease Institute & Centre for Primary Care, University of Antwerp, Belgium; 2 Department of General Practice and Primary Health Care, Ghent University, Belgium; 3 Department of Psychiatry, Ghent University Hospital Belgium; 4 Department of Geriatrics, Ghent University, Belgium; 5 Heymans Institute for Pharmacology and Pharmacotherapy, Ghent University, Belgium

## Abstract

**Background:**

Polypharmacy is common among older people. The purpose of this study is to describe GPs' views and beliefs on polypharmacy in order to identify the role of the GP in relation to improving prescribing behaviour. The awareness of these often established beliefs is key for understanding behaviour and promoting change which can guide action towards more rational prescribing.

**Methods:**

A qualitative descriptive methodology was used with semi-structured interviews. Interviews were conducted with 65 GPs from the region of Aalst, a district of a mixed urban and rural population in Belgium. The aim of the study was to describe the GPs' perspectives on polypharmacy in primary care.

**Results:**

GPs acknowledge that polypharmacy is a problem in their older patient population, especially because of the risk of adverse drug reactions, interactions and lowered adherence. GPs mention that difficulties in keeping an overview of the exact medication intake is an important problem caused by polypharmacy. The patients' strong belief in their medication and self-medication are seen as important barriers in reducing the number of drugs taken. Next to these patient related factors, there are some factors related to the prescriber, such as the lack of regular evaluation of the medication schedule by GPs and the involvement of several prescribers, especially in a hospital setting. According to the respondents, prevention and evidence based medicine guidelines often induce polypharmacy.

**Conclusions:**

GPs point out that polypharmacy is an important problem in their older patient population. They see an important role for themselves in optimizing drug regimens for their patients. However, they do not have a readymade solution for polypharmacy. The limited set of options for addressing polypharmacy leave GPs feeling powerless to tackle the problem. There is a need for simple GP friendly tools and access to pharmacotherapeutic advice. Future research in this area and interventions seeking to improve prescribing for the elderly will have to focus on practical tools and take into account the GPs' sense of helplessness.

## Background

### Polypharmacy

Prescribing medication is becoming more difficult and complex. The inherent risk of adverse reactions and interactions rises because of the pharmacological complexity of modern drugs, the ageing population, and the increasing polypharmacy [1]. Polypharmacy can be defined as the concomitant use of 3 or more drugs [2] or the use of more drugs than indicated. Polypharmacy is particularly common among older adults - around 20% of people over 70 in the Western World are taking five or more drugs [3]. Although appropriate medication can reduce symptoms, morbidity and mortality in older patients, drugs can also be dangerous. Polypharmacy increases the risk of having adverse drug reactions and/or interactions, it also means that unnecessary drugs may be obscured by the large number of necessary ones [4-7]. The risk for reduced adherence to the prescribed regimen increases as the number of prescribed medications increases [8,9]. In older adults, between 10% and 20% of hospital admissions are drug related [5].

### The role of the general practitioner

Even though the general practitioner (GP) is not always the prescriber, most patients have a longstanding relationship with their GP. Moreover, the GP is ideally placed to have a global overview of the medication intake of his/her patient. Many quantitative studies have identified deficiencies in the use and prescription of medication, yet, there are few qualitative investigations on the views of general practitioners (GPs) on polypharmacy. Understanding processes and mechanisms of underlying behaviour is vital in achieving behaviour change [10].

### Purpose of the study

The purpose of this study is to describe GPs' views and beliefs on polypharmacy in order to identify the role of the GP in relation to improving prescribing behaviour. The awareness of these often established beliefs is one of the most important drivers for change as it can guide the action needed for more rational prescribing.

## Methods

### Study design

A qualitative descriptive methodology [11] was used to explore the views of GPs on polypharmacy in order to establish a comprehensive summary of events in every-day terms with the aim of developing a description of the views of GPs towards polypharmacy.

Individual semi-structured interviews were used to explore the perspectives of general practitioners, as they are particularly useful when discussing sensitive issues like polypharmacy. Data collection by semi-structured interviews allows participants to be asked questions within a flexible framework [12]. The study was approved by the ethics committee of the Ghent University Hospital and written informed consent was obtained from all respondents.

### Participants

The study was conducted in the district of Aalst (a city of 80 000 inhabitants with two hospitals; a poor central population and rich residential quarters as well as rural residents). All 102 GPs from a list of GPs from Aalst were contacted by letter and invited to participate in the study. After a week they were contacted by telephone and 65 GPs agreed to be interviewed at their practice. The purposeful maximum variation sample [13] of 65 general practitioners (40 men and 25 women) with an average age of 50 years reflected a wide variety in terms of experience and location (city and rural).

The research team developed a semi-structured interview guide after a preliminary review of the literature. The interview guide consisted of the following broad topic sections: reflections on polypharmacy in general practice; factors contributing to polypharmacy in general practice; reflections on GPs' specific role and their attitudes to interventions to optimize prescribing. Within each broad section of the interview topic guide there were more detailed questions and specific probes to allow the discussion to develop (Table 1). After each interview, the interviewers completed a debriefing to discuss relevant contextual information, general impressions of the interview, and possible changes to the interview guide. Audiotapes of each interview were transcribed verbatim.

**Table 1:** Interview Questions

Broad Questions
What are your views on polypharmacy in general practice?
In your opinion, can something be done in order to reduce polypharmacy?
What could be a specific role for a GP in relation to polypharmacy and prescribing?
**Specific Questions and Prompts**
What are important factors contributing to polypharmacy?
Are there negative things about polypharmacy?
Do you think prescribing practices have changed over time?
Are there specific barriers that you can think of in order to reduce polypharmacy?
Are there specific elements that could help reduce polypharmacy? (types of interventions, education, ...)

### Data analysis

We used a qualitative content analysis [14,15] which is a good method in qualitative descriptive methodology [11] orientated towards summarizing the informational content of the data. The interviews were read by two researchers (SA & AT) to become familiar with the data. The text about the views of the GPs on polypharmacy was then extracted by two independent coders and brought together as a unit of analysis and labelled with different codes by first highlighting the exact words from the text that appeared to capture key thoughts. The various codes were compared and sorted into categories. Finally, the underlying meaning was formulated into themes after discussion with the different research members.

### Rigour of the study

The research team used several methods to ensure the study was rigorous and trustworthy. Audio-taped and written verbatim versions of the interviews were compared to ensure accuracy and completeness of data. Debriefing and field notes were maintained and reviewed by team members.

## Results

On the whole GPs acknowledge that polypharmacy is a problem in their older patient population and a major challenge for general practice. The data show that the respondents identify 4 themes that influence polypharmacy, namely patient related, GP related, evidence based medicine (EBM) and specialist related factors.

### Patient related factors influencing polypharmacy according to the GPs

Being the major consumers, older adults are particularly vulnerable to adverse drug reactions.

"With older age, you have to take into account that organs are not working properly anymore." (36) "The chance of side effects of medication in this older age group is also much higher: kidney function is not as good anymore and then you start treating side effects..." (20)

Side effects are not always recognized as such. They can be very pronounced in this population and this can lead to a pharmacological treatment of side effects or even to hospital admission.

GPs acknowledge the difficulty in keeping an overview of the exact intake of medication of their older patients. When older adults have to take a lot of drugs, the danger of self medication exists - patients change their own regimens by discontinuing them, lowering, increasing or skipping doses without consulting their GP.

"...sometimes the older people decide for themselves to reduce some of their medication or to adjust the doses without telling their GP. Therefore as their GP you can have the wrong impression about their medication intake..." (28)

The risk of this is that GPs prescribe additional drugs as it seems the previous doses are not having the expected effect. Therefore GPs place a lot of emphasis on the importance of compliance.

Patients are not always inclined to stop using drugs that they have used for a long time. Some patients can be demanding and difficult when their use of medication is questioned and resist any attempt to change their prescriptions:

"A lot of medication that has once been prescribed is being taken daily. The patient feels fine and does not want to change the medication regime" (38)

Most GPs recognize a very strong attachment in many patients to benzodiazepines or pain medication whilst they also acknowledge that these are some of the drugs that should be avoided or reduced.

GPs perceive self-medication as a real problem. The GP is not the only provider, the older people often receive medication from friends, relatives or from neighbours. They do not perceive this as their prescribed medication and consequently, they do not take into account the possible side effects or interactions:

"They take a blood-diluting drug. Then they take another aspirin with another brand name because their neighbour told them that they should take one daily. They have no idea what they are taking..." (31)

### The role of GP related factors and its influence on polypharmacy & suggested solutions

GPs refer to polypharmacy as a slowly growing process and because of that they do not pay sufficient attention to this phenomenon. It is easy to start a new treatment for every new complaint without really evaluating the existing medication schedule. They do find that they are not critical enough. This routine approach might be one of the factors that make polypharmacy so common. Patients keep on taking their prescribed medication (they feel good about it), whilst none is stopped or re-evaluated:

"The number of medications grows slowly. There is a complaint, we give new medication, it continues without really stopping it after a while...and it is our responsibility to try and withdraw it from the patient..."(43)

GPs feel strongly that their role is as a 'gatekeeper' whose responsibility is to control the type and quantity of medication used. They mention that established routines for assessing the total medication schedule of the patient are necessary.

GPs feel they should take the initiative to prescribe only the essential medication and to lower doses. They also realise it is not necessary to treat every single symptom but to look at the overall health status of their patient and the quality of life. GPs suggest that a list of priority medication in order of importance might be very helpful. The respondents mention that it is essential to be alert at every single consultation. For every prescription renewal they should ask themselves whether the medication is still necessary and if not they should stop the prescribed medicine.

### The role of evidence based medicine in polypharmacy according to the GPs

GPs feel under pressure from guidelines to prescribe preventive drugs, even though the negative impacts of polypharmacy may outweigh the possible benefits from individual drugs:

*"If you want to follow the evidence based guidelines then you need to work preventively. Then quickly you will come to six additional medications..."(36) " If you look at the guidelines on what to prescribe post-infarct...Strictly speaking that is 6 additional drugs...You will have to draw a line between what is scientifically proven and what is realistic in daily practice..." (49)*.

GPs are aware of the increased risk of interactions. They should be alert to this but they admit it is difficult to keep an overview when there is polypharmacy. GPs report difficulties in differentiating between medical conditions or symptoms due to side effects of medication.

They also experience shortcomings in their pharmacological knowledge. The information available is not always that accurate or up-to-date:

"We do not always have an overview of the interactions, that is a big problem, firstly there is not much information available for us GPs, and secondly when there is information available to us it does not mean that we know it; it is not that simple..." (26)

### The role of increased specialization in health care and its influence on polypharmacy

One of the reasons why GPs find polypharmacy a complicated issue is that often more than one prescriber is involved. Inappropriate prescribing can arise from the absence of communication between doctors practising in different settings or even between specialists practising in the same setting. Older adults often have several chronic conditions and need several drugs; they are often admitted to hospital and should have regular reviews of their treatment.

GPs see a role for themselves to protect the patient through regular follow-up.

"The GP has a holistic view of the patient. A specialist often does not have the time to speak with the patient about the entire medication regime. The GP can contact the specialist...The coordination of the medication regime of different disciplines is a tough job..." (12)

When the patient has been admitted to hospital it is important to re-evaluate the medication schedule. GPs find it important to have a coordinating role. They have a holistic view of the patient because of the long standing doctor-patient relationship. This is in contrast to a specialist who only looks at the patient from his or her own discipline. This is perceived as a very tough job for GPs with major implications for their workload:

"As a GP you have a broader view of your patient. You look at him/her from his own life. Specialists narrow the things down a little bit. I think it's very important that there is one coordinator who watches out over the patient and sees that the pneumologist does not prescribe something that can affect the cardiologic state of the patient...." (41)

## Discussion

This study highlights some of the GPs' perceptions and beliefs on polypharmacy. The perceptions of this important group of prescribers on one of the main problem areas contributing to polypharmacy have not been investigated until now. As the respondents in our study remark, the primary care setting is seen as ideal for addressing the problem of inappropriate prescribing, this is in concordance with the literature [16]. Primary care physicians are ideally placed to optimize drug regimens given their knowledge of patient-specific information and their ability to coordinate the patient's overall medical care [16].

Despite the GPs' recognition that polypharmacy is a problem in their older patient population and the fact that GPs perceive it as their role in addressing the problem, they feel largely helpless to tackle it. GPs do not have a 'ready-made' solution for polypharmacy.

It is important to point out that GPs experience obstacles at different levels. One of the barriers is at the level of the patient. The GPs believe that patients have a joint responsibility. In their view the best option to achieve medication reduction is to reduce drugs that are used without a clear indication. Patients on the other hand are not always inclined to stop taking medication that they have been using chronically [17]. This is a well known problem as for example in regard to other drugs such as hypnotics [18]. Improvement of knowledge alone may not solve the problem for GPs. They need skills and tools to strengthen their confidence as GPs perceive it as difficult to motivate patients to withdraw from their medication. Motivational strategies may be an important educational tool for GPs or community nurses and even for pharmacists.

Medication compliance is inversely correlated with number of drugs taken [8,9]. According to the GPs' opinion compliance and stimulation of compliance is one of the most important challenges.

GPs also point out that older people are particular vulnerable to adverse drug reactions, which are often preventable [19]. Our participants do not refer to specific strategies to reduce adverse drug effects, however other researchers have designed screening tools to detect prescribing that is potentially inappropriate. Examples of such screening tools are the Beers' criteria [20], the improving prescribing in the elderly, the screening tool to help guide doctors towards the right treatment (START) [19], NO TEARS [21] and ACOVE [22]. Unfortunately these tools are not very useful or practical for routine clinical screening as they are too time consuming and too complex. Further research is underway for new tools that are easier to use in primary care [19]. There is a need for simple approaches such as working in close collaboration with skilled pharmacists or peers for 'medication review'[23].

Another barrier is at the level of management. GPs also talk about their own contribution to polypharmacy. They find that they are often not critical enough when starting a new treatment. They see an important role for themselves in controlling the type and quantity of medication used.

Many respondents bring up EBM guidelines as something which encourages polypharmacy. This is also described in the literature [18,24,25]. A combination of different drugs is often suggested as the 'golden standard' for treating a disease. There is more and more emphasis on giving medication as a way of preventing diseases. For older people preventive aims are often minimal considering their age and polypathology, which is in contrast with guidelines talking about one specific disease. The disadvantage sometimes does not outweigh the gain of taking medication.

The respondents in this study mention a lack of pharmacological knowledge. There is a lack of adequate training of doctors in geriatric pharmacotherapy [26]. In literature several strategies have been tested to optimise prescribing such as educational approaches, computer assisted approaches, a medication review by clinical pharmacists, geriatric medicine services, multidisciplinary approaches and multifaceted approaches [23,27]. Yet, despite the substantial resources devoted to developing and testing the effectiveness of interventions to improve prescribing, widespread diffusion of successful methods has not yet been achieved [23]. When aiming at setting up interventions for GPs, one has to be aware that updating knowledge and information alone is not enough. There is a need for a change in attitude and behaviour of GPs and they need tools for this.

Most prescribing is done by the patient's GP, but medication is often started or adjusted in secondary care. In Belgium and in other European countries specialists can work relatively independently without referral from GPs [28]. A recent study in the US found that the incidence of adverse drug reactions is directly related to the number of doctors prescribing [29]. The findings also showed the reluctance of GPs to interfere with treatment prescribed by a colleague as one of the reasons mentioned for polypharmacy this has also been found in previous research [26]. Therefore good communication between GP and hospital, GP and patient and GP and carers is crucial [7].

## Conclusions

GPs point out that polypharmacy is an important problem in their older patient population. They see an important role for themselves in optimizing drug regimens for their patients. However, they do not have a readymade solution for polypharmacy. The limited set of options for addressing polypharmacy leave GPs feeling powerless to tackle the problem. There is a need for simple GP friendly tools and access to pharmacotherapeutical advice. Future research in this area and interventions seeking to improve prescribing for the elderly will have to focus on practical tools and take into account the GPs' sense of helplessness.

## Competing interests

The authors declare that they have no competing interests.

## Authors' contributions

SA, AT MP, TC were involved in discussion that led to the original idea of the research. AT collected the data. SA and AT were involved in analysing the data and writing the first draft of the paper. All authors critically revised the paper for important intellectual content and approved of the final version.

## Pre-publication history

The pre-publication history for this paper can be accessed here:

http://www.biomedcentral.com/1471-2296/11/65/prepub
